# Potential Impact of the Z0011 Trial on the Omission of Axillary Dissection: A Retrospective Cohort Study

**DOI:** 10.1055/s-0041-1725052

**Published:** 2021-03-30

**Authors:** Julia Yoriko Shinzato, Katia Piton Serra, Caroline Eugeni, Cesar Cabello, Cassio Cardoso-Filho, Luís Carlos Zeferino

**Affiliations:** 1 Gynecology and Obstetrics Department, Universidade Estadual de Campinas, Campinas, SP, Brazil; 2 Gynecology and Obstetrics Department, Faculdade São Leopoldo Mandic, Campinas, SP, Brazil

**Keywords:** breast cancer, sentinel lymph node biopsy, lymph node dissection, axilla, clinical trial, câncer de mama, biópsia do linfonodo sentinel, dissecção de linfonodos, axila, estudo clínico

## Abstract

**Objective** To evaluate the number of patients with early-stage breast cancer who could benefit from the omission of axillary surgery following the application of the Alliance for Clinical Trials in Oncology (ACOSOG) Z0011 trial criteria.

**Methods** A retrospective cohort study conducted in the Hospital da Mulher da Universidade Estadual de Campinas. The study population included 384 women diagnosed with early-stage invasive breast cancer, clinically negative axilla, treated with breast-conserving surgery and sentinel lymph node biopsy, radiation therapy, chemotherapy and/or endocrine therapy, from January 2005 to December 2010. The ACOSOG Z0011 trial criteria were applied to this population and a statistical analysis was performed to make a comparison between populations.

**Results** A total of 384 patients underwent breast-conserving surgery and sentinel lymph node biopsy. Of the total number of patients, 86 women underwent axillary lymph node dissection for metastatic sentinel lymph nodes (SNLs). One patient underwent axillary node dissection due to a suspicious SLN intraoperatively, thus, she was excluded from the study. Among these patients, 82/86 (95.3%) had one to two involved sentinel lymph nodes and met the criteria for the ACOSOG Z0011 trial with the omission of axillary lymph node dissection. Among the 82 eligible women, there were only 13 cases (15.9%) of lymphovascular invasion and 62 cases (75.6%) of tumors measuring up to 2 cm in diameter (T1).

**Conclusion** The ACOSOG Z0011 trial criteria can be applied to a select group of SLN-positive patients, reducing the costs and morbidities of breast cancer surgery.

## Resumo

**Objetivo** Avaliar o número de pacientes com câncer de mama em estágio inicial que se beneficiariam da omissão da linfadenectomia axilar segundo o protocolo Z0011 da *Alliance for Clinical Trials in Oncology* (ACOSOG).

**Métodos** Estudo de coorte retrospectiva conduzido no Hospital da Mulher da Universidade Estadual de Campinas. Foram incluídas mulheres diagnosticadas com carcinoma invasivo de mama em estágio inicial, com axila clinicamente negativa, tratadas com cirurgia conservadora e biópsia do linfonodo sentinela, radioterapia, quimioterapia e/ou hormonioterapia, de janeiro de 2005 a dezembro de 2010. Os critérios do estudo da ACOSOG Z0011 foram aplicados a essas mulheres e foi realizada uma análise estatística que comparou ambas as populações dos estudos.

**Resultados** Foram estudadas 384 mulheres submetidas a cirurgia conservadora de mama e biópsia do linfonodo sentinela. Entre elas, 86 mulheres foram submetidas a linfadenectomia axilar por metástase presente no linfonodo sentinela. Uma paciente foi submetida a linfadenectomia axilar por ter um linfonodo palpável suspeito no intraoperatório, não incluída no estudo. Entre essas 86 pacientes, 82 (95,3%) tiveram de 1 a 2 linfonodos sentinela comprometidos e seriam elegíveis para omissão da linfadenectomia axilar pelos critérios do ACOSOG Z0011. Entre as 82 pacientes elegíveis, apenas 13 (15,9%) delas apresentaram tumores com invasão angiolinfática, e 62 (75,6%) dos tumores mediram até 2 cm (T1).

**Conclusão** Os critérios do estudo ACOZOG Z0011 podem ser aplicados a um seleto grupo de pacientes com linfonodo sentinela positivo reduzindo os custos e a morbidade cirúrgica do tratamento do câncer de mama.

## Introduction

The recommendations for axillary management in breast cancer have changed rapidly over the years with advances in surgical techniques and scientific knowledge.

Sentinel lymph node biopsy (SLNB) in breast cancer with clinically negative axilla has currently been included in staging protocols in the majority of referral centers for breast cancer treatment. Axillary lymph node dissection (ALND) still has a major role in locoregional disease control. Furthermore, in case of positive sentinel lymph nodes (SLNs), it was the standard of practice, a fact that has been modified with new studies. 1 2 3 4 5 6

Various studies have attempted to determine factors associated with a higher chance of additional lymph node involvement in the remaining axilla after a positive SLNB. The intent is to help decide which patients should undergo ALND, thus avoiding more invasive surgeries in patients who would fail to derive any benefit. 7

Nevertheless, recent research has shown that ALND can be avoided in many cases, even in patients with positive SLN. In these cases, surgery could be replaced by radiotherapy either directly or tangentially to the axillary drainage chain, reducing further morbidity and sequelae of surgical treatment, without affecting patient prognosis. 8 9 10

The ACOSOG Z0011 trial evaluated women with invasive breast carcinoma who had tumors measuring up to 5 cm (T1 and T2), clinically negative axilla, and who were undergoing breast-conserving surgery (BCS). All patients received proper radiotherapy and systemic adjuvant therapy. Women who had up to 2 metastatic axillary lymph nodes at the time of SLNB were randomized to ALND or received no complementary axillary therapy. The study showed that there was no difference in overall survival and disease-free survival between groups. 8

The aim of the After Mapping of the Axilla: Radiotherapy or Surgery (AMAROS) trial was to compare ALND versus axillary radiotherapy. It evaluated women with invasive breast carcinoma, tumors measuring up to 5 cm (T1 and T2), clinically negative axilla, undergoing BCS or mastectomy. Those with positive SLN were randomly assigned to ALND or axillary radiotherapy. Both groups achieved good tumor control at the 5-year follow-up. However, the group undergoing radiotherapy to the axilla had less morbidity, mainly resulting from lymphedema. 9

Some centers have already adopted a conservative approach to the axilla based mainly on the ACOSOG Z0011 and AMAROS trials.

The aim of the current study was to apply the ACOSOG Z0011 trial criteria to women undergoing breast cancer treatment in the Hospital da Mulher Prof. Dr. José Aristodemo Pinotti - Centro de Atenção Integral à Saúde da Mulher (CAISM, in the Portuguese acronym) and investigate the number of women who could be spared from ALND.

## Methods

The current study is part of a retrospective cohort study which was approved by the Research Ethics Committee of the University of Campinas School of Medicine, CAAE 36001314.4.0000.5404, under number 839.129. Data collection was conducted using medical records of invasive breast cancer patients managed in the Hospital da Mulher Prof. Dr. José Aristodemo Pinotti - CAISM from January 2005 to December 2010. A total of 501 patients, with T1 and T2 breast cancer and clinically negative axilla and without neoadjuvant treatment, underwent mastectomy or breast cancer surgery (BCS) always followed by SLNB. Out of the total number, 384 were selected for BCS with SLNB. The SLN was identified by injection of radioactive technetium colloid and subsequent lymphoscintigraphy of the breast or by injection of patent blue dye in an isolated or combined technique. The SLNs were evaluated in frozen tissue section examination by touch imprint cytology. When positive, diagnosis was further confirmed by histologic frozen section examination. When negative, the surgeon awaited the results of paraffin tissue section and, if positive, surgery would proceed with total ALND. Immunohistochemistry was performed if the paraffin tissue section (from histologic frozen section) was free of cancer. Women who had lymph node involvement in the paraffin tissue section, such as macro- or micrometastases, later underwent ALND, except when micrometastases or isolated tumor cells (ITC) were detected on immunohistochemistry. All women had clear surgical margins in the first surgery or in further surgeries to widen the incision and later received adjuvant treatment according to institutional protocol and radiotherapy to the remaining breast with a dose of 50 Gy and radiation boost with 10 Gy to the surgical scar. The anatomical field limits of the breast are defined superiorly by the second intercostal space; inferiorly, it is located around 1 cm below the breast; at the medial limit, on the parasternal line; and laterally, on the middle axillary line. The ipsilateral supraclavicular fossa was part of the irradiation field in case of positivity due to macrometastases of axillary lymph nodes in any number.

### Statistical Analysis

To describe the sample profile according to the variables studied, tables of frequencies of categorical variables, and descriptive statistics of numerical variables were constructed. To compare categorical variables between groups, the chi-square or Fisher exact test was used. To compare numerical variables, the Mann-Whitney test was used. The level of significance adopted for the statistical tests was 5%, that is, *p* < 0.05. The software package used was SAS, version 9.2 (SAS Institute Inc., Cary, NC, USA.

## Results

**Fig. 1:**
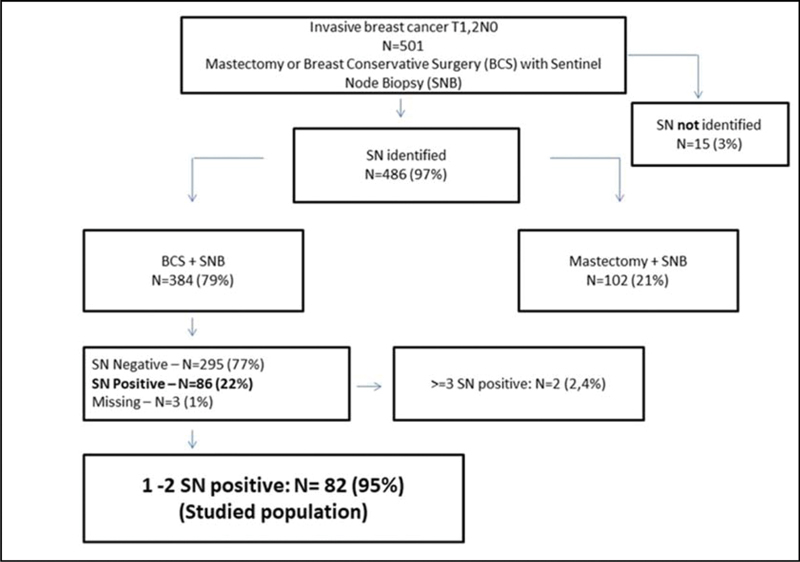
Sample selection.

Among 501 selected patients, 102 underwent mastectomy and 384 received BCS. Sentinel lymph node was identified in 486 cases. Out of the patients undergoing BCS, 295 were SLN-negative and 86 were SLN-positive. Axillary lymph node dissection was conducted in 87 patients (86 with SLN involvement and one with a macroscopically suspicious node during surgery, which was excluded from the study [ Fig. 1 ]). Concerning the number of involved SLNs, 82/86 (95.3%) of the women had 1 or 2 positive SLNs (study population), 1 had 3 involved SLNs (1.2%), and 1 had 4 (1.2%) or more involved SLNs ( Table 1 ).

**Table 1:** Number of involved lymph nodes

Number of positive SLNs	N = 86	Percentage (%)
1	70	81.4
2	12	13.9
3	1	1.2
≥ 4	1	1.2
Missing	2	2.3

**Abbreviations: N, number; SLN, sentinel lymph node.**

The clinical and pathological characteristics are described in Table 2 . The majority of the patient population was over 50 years of age, with a mean of 56 years in the total population and 55 years in the studied population. Most tumors were invasive carcinomas of no special type (invasive ductal carcinoma), which measured up to 1 cm in diameter, expressed estrogen and/or progesterone receptors, did not overexpress HER2, and had no lymphovascular invasion.

**Table 2:** Clinicopathological features of treated patients

Characteristics	Total sample ( *n* = 384)	Studied population ( *n* = 82)	Complementary ( *n* = 302)	*p* -value
Age (yr)				
Average	56	55	56	0.363
Missing	0	0	0	
Age (yr)				
≤ 50, N (%)	120 (31.3)	29 (35.4)	91 (30.1)	0.365
> 50, N (%)	264 (68.7)	53 (64.6)	211 (69.9)	
Missing	0	0	0	
Clinical T stage, N (%)				
T1	300 (78.1)	62 (75.6)	238 (78.8)	0.715
T2	75 (19.5)	19 (23.2)	56 (18.5)	
T3	6 (1.6)	1 (1.2)	5 (1.6)	
Missing	3 (0.8)	0	3 (1.1)	
Receptor status, N (%)				
ER+ and/or PgR+	318 (82.8)	75 (91.5)	243 (82.1)	0.040
ER-/PgR-	60 (15.6)	7 (8.5)	53 (17.9)	
Missing	6 (1.6)	0	6	
HER2 status, N (%)				
HER2 +	59 (15.4)	10 (12.2)	49 (16.2)	0.408
HER2 -	182 (47.4)	40 (48.8)	142 (47)	
Missing	143 (37.2)	32 (39)	111 (36.8)	
LVI, N (%)				
Yes	34 (8,8)	13 (15.9)	21 (6.9)	0.012
No	349 (90.9)	69 (84.2)	280 (92.7)	
Missing	1 (0.3)	0	1 (0.4)	
Tumor type, N (%)				
Infiltrating ductal	317 (82.5)	66 (80.5)	251 (83.1)	0.314
Infiltrating lobular	13 (3.4)	5 (6.1)	8 (2.6)	
Other	48 (12.5)	10 (12.2)	38 (12.6)	
Missing	6 (1.6)	1 (1.2)	5 (1.7)	

Abbreviations: LVI, lymphovascular invasion; N, number.

Table 3 compares the study population with patients who had been spared from axillary surgery in the ACOSOG Z0011 trial (up to 2 positive SLNs). There was no difference in patient age between the groups. No difference was observed in tumor type and expression of hormone receptors and HER2. Nevertheless, in our population, there was a higher number of patients with tumors measuring up to 2 cm ( *p* = 0.039) and fewer cases of lymphovascular invasion ( *p* < 0.001), presenting a larger case study of tumors with a more favorable prognosis.

**Table 3:** Clinicopathological features of studied population versus ACOSOG Z0011

Characteristics	Studied population ( *n* = 82)	Z0011 trial ( *n* = 420)	*p* -value
Age (yr)			
Average	55	56	0.567
Missing	0	7	
Age (yr)			
≤ 50, N (%)	29 (35.4)	135 (32.1)	0.638
> 50, N (%)	53 (64.6)	278 (66.2)	
Missing	0	7 (1.7)	
Clinical T stage, N (%)			
T1	62 (75.6)	284 (67.6)	0.039
T2	19 (23.2)	134 (31.9)	
T3	1 (1.2)	0	
Missing	0	2 (0.5)	
Receptor status, N (%)			
ER+ and/or PgR+	75 (91.5)	320 (76.2)	0.069
ER-/PgR-	7 (8.5)	63 (15)	
Missing	0	37 (8.8)	
LVI, N (%)			
Yes	13 (15.9)	129 (30.7)	<0.001
No	69 (84.2)	189 (45)	
Missing	0	102 (24.3)	
Tumor type, N (%)			
Infiltrating ductal	66 (80.5)	344 (81.9)	0.921
Infiltrating lobular	5 (6.1)	27 (6.4)	
Other	10 (12.2)	45 (10.7)	
Missing	1 (1.2)	4 (1)	

**Abbreviations:** ACOSOG, Alliance for Clinical Trials in Oncology; LVI, lymphovascular invasion; N, number.

## Discussion

The aim of the present study was to reproduce the ACOSOG Z0011 trial criteria in 384 women with early-stage T1 to T2 invasive breast cancer with clinically negative axilla, undergoing BCS and SLNB. Out of the total number of women, 87(22.6%) underwent ALND, and 297 (77.3%) were spared from further treatment. If we had applied the ACOSOG Z0011 trial criteria, 82/86 (95.3%) more women might have benefitted from the omission of ALND, corresponding to a total of 377 women spared from axillary surgery, that is, 98.2% of all women undergoing SLNB (295 had negative SLN).

In 2016, Verheuvel et al. 11 investigated 916 cases undergoing ALND for SLN involvement or positive axillary lymph node diagnosed by ultrasound-guided biopsy. Of the total number of patients, 558 (61%) could have benefitted from the omission of ALND. Those authors considered micrometastases and isolated tumor cells (ITCs) as N0. In 2013, Delpech et al. 12 applied the same criteria to 125 SLN-positive patients undergoing ALND. Among those women, 87 (69.7%) were potentially eligible for omission of ALND. 11 In our study, we found an even higher number of cases in which ALND could have been avoided.

The preestablished concept of ALND in all patients with SLN involvement has currently undergone modifications. When the SLN technique emerged in the late 90s, it was a major advance for women with negative axilla, who would no longer require ALND. Nevertheless, SLN-positive patients still received axillary dissection. Axillary lymph node dissection may cause complications, such as postoperative seroma, infection, sensory disturbances in the ipsilateral arm in the medium and long-term, in addition to lymphedema in up to 40% of cases at the 10-year follow-up. 13 Complications after ALND interfere negatively in the quality of life of these women, increasing treatment expenses. 14

In 2014, Sackey et al. 15 compared a group of women undergoing SLNB alone to another group undergoing ALND due to positive SLN and found a significantly lower risk of lymphedema in women who had not received ALND. In 2013, in a long-term follow-up study, De Gournay et al. 16 failed to find any case of lymphedema in the SLNB group, while lymphedema rates were 10.3% in the ALND group and 7% in the SLNB group, followed by ALND. 15 16 It can be inferred that the omission of ALND in a public health care facility of a developing country, such as Brazil, could reduce the cost of surgical treatment and management of potential sequelae related to ALND. Furthermore, it could promote a better quality of life in a large number of women by reducing the possibility of lymphedema.

Lymph node involvement in the remaining axilla ranges from 20 to 40% in SLN-positive patients. 1 2 3 In the recent past, studies have attempted to correlate predictive factors for lymph node involvement in the remaining axilla. The Memorial Sloan-Kettering Cancer Center (MSCC) created a nomogram using factors correlated with the tumor size, histologic type, nuclear grade, lymphovascular invasion, multifocality and estrogen receptor, thus screening a group of SLN-positive women who might benefit from the omission of ALND. 3 Several attempts have been made to reproduce and validate the MSCC nomogram, with conflicting results. 17 18

Currently, both locoregional and distant disease control have improved by systemic therapy, allowing for less extensive axillary surgery. Studies have demonstrated that even with the potential persistence of disease in the remaining axilla, regional recurrence rates have not corresponded to these possibilities. 10

Radiation therapy after BCS using tangential fields to the axilla in the ACOSOG Z0011 trial probably covers the remaining positive axillary nodes. 19 The AMAROS trial also showed that radiation therapy had promising results in local disease control as well as in comorbidities, lymphedema in particular. 9 Thus, regional axillary treatment with surgery or even radiation therapy, omitted in some select cases, may confer benefits in the quality of life and reduce treatment costs.

The novelty of the ACOSOG Z0011 trial lies in the demonstration of good outcomes without any further treatment of the remaining axilla in women with up to 2 involved SLNs. 19

The current study has some limitations. This is a retrospective study, and, initially, in our center it was routine practice to consider the presence of micrometastases and ITC as a SLN-positive axilla. Over time, there was a change in concept, and this practice was abandoned. Therefore, a possible explanation for such a high number of ALND that could have been avoided is the number of patients with micrometastases and ITC. Currently considered N0, these patients were entered in the case study of N1 and underwent ALND at the time. Although this paper does not present innovation on the axillary role for the “ACOSOG Z0011-like” population, it was the local reality 5 years before the Z0011 protocol was accepted by our Breast Unit Committee. Nowadays, this protocol has become the gold-standard treatment of early-stage breast cancer (cT1–2 cN0 cM0) in our institution, as well as in all breast treatment reference centers in Brazil. It is worth pointing out that these data were coming from a public university institution, and the total costs of procedures should be accepted and funded by the Brazilian National Health System (SUS). Unfortunately, we couldn't obtain data about lymphedema. These data could have improved the study.

In addition to reproducing the ACOSOG Z0011 trial criteria, the present study showed a paradigm shift in axillary treatment over the years. It enabled us to make a critical evaluation of our routine practice, which is no different from other reference centers worldwide. 11 12 Self-criticism is fundamental for program implementation to provide patients with the best treatment, along with the least associated comorbidities and lowest cost possible, since this is a public health care facility in a developing country.

## Conclusion

The ACOSOG Z0011 trial criteria can be applied to a select group of SLN-positive patients, reducing the costs and morbidities of breast cancer surgery.
